# Enhanced non-linear optical properties of porphyrin-based polymers covalently functionalized with graphite phase carbon nitride

**DOI:** 10.3389/fchem.2022.1102666

**Published:** 2022-12-15

**Authors:** Chen Liang, Xu Cui, Wenyue Dong, Jieming Qin, Qian Duan

**Affiliations:** ^1^ School of Materials Science and Engineering, Changchun University of Science and Technology, Changchun, China; ^2^ Engineering Research Center of Optoelectronic Functional Materials, Ministry of Education, Changchun, China

**Keywords:** porphyrin-based polymer, graphite phase carbon nitride, nanohybrids, non-linear optical (NLO) materials, optical limiting

## Abstract

In our work, a flurry of original porphyrin-based polymers covalently functionalized g-C_3_N_4_ nanohybrids were constructed and nominated as PPorx-g-C_3_N_4_ (x = 1, 2 and 3) through click chemistry between porphyrin-based polymers with alkyne end-groups [(PPorx-C≡CH (x = 1, 2 and 3)] and azide-functionalized graphitic carbon nitride (g-C_3_N_4_-N_3_). Due to the photoinduced electron transfer (PET) between porphyrin-based polymers [PPorx (x = 1, 2 and 3)] group and graphite phase carbon nitride (g-C_3_N_4_) group in PPorx-g-C_3_N_4_ nanohybrids, the PPorx-g-C_3_N_4_ nanohybrids exhibited better non-linear optical (NLO) performance than the corresponding PPorx-C≡CH and g-C_3_N_4_-N_3_. It found that the imaginary third-order susceptibility (*Im* [χ^(3)^]) value of the nanohybrids with different molecular weight (MW) of the *p*Porx group in the nanohybrids ranged from 2.5×10^3^ to 7.0 × 10^3^ g mol^−1^ was disparate. Quite interestingly, the *Im* [χ^(3)^] value of the nanohybrid with a *p*Porx group’s MW of 4.2 × 10^3^ g mol^−1^ (PPor2-g-C_3_N_4_) was 1.47 × 10^–10^ esu, which exhibited the best NLO performance in methyl methacrylate (MMA) of all nanohybrids. The PPorx-g-C_3_N_4_ was dispersed in polymethyl methacrylate (PMMA) to prepare the composites PPorx-g-C_3_N_4_/PMMA since PMMA was widely used as an alternative to glass. PPor2-g-C_3_N_4_/PMMA showed the excellent NLO performance of all nanohybrids with the *Im* [χ^(3)^] value of 2.36 × 10^–10^ esu, limiting threshold of 1.71 J/cm^2^, minimum transmittance of 8% and dynamic range of 1.09 in PMMA, respectively. It suggested that PPorx-g-C_3_N_4_ nanohybrids were potential outstanding NLO materials.

## 1 Introduction

Non-linear optical (NLO) materials have received great attention owing to their tremendous applications in the fields of photonic devices (Andréasson et al., 2011), optical limiting (Wang et al., 2019), optical switches (Wang C. et al., 2021), light converters (Miriyala and Mani, 2021), *etc.* Porphyrins with a unique 18 π electron conjugated structure, good thermal stability and narrow bandgap possess excellent NLO performance (Manjunatha et al., 2020; Ou et al., 2021). Meanwhile, due to the structural adjustability, unique electronic and photophysical properties of porphyrins, porphyrins have broad application in various areas and good prospects (Zhang et al., 2018; Chen et al., 2020; Asghar et al., 2021; Liu and Cheng, 2021). Particularly, the NLO performance of porphyrins could be improved by the flexible modification of peripheral substituents or the hybridization with other materials (Zawadzka et al., 2013; Woller et al., 2016; Hu et al., 2018; Biswal et al., 2019; Samal et al., 2019; Liu J. L. et al., 2020; Liu Z. et al., 2020; Liu and Cheng, 2021; Ramasamy et al., 2022). However, previous research indicated the self-aggregate behavior of porphyrins could lead to the formation of large macro-scale and fractal structures, causing a negative impact in the development of NLO devices in practice (Kalachyova et al., 2014). Therefore, the development of novel porphyrin-based NLO materials remained challenging, which have become one of the hot issues in the NLO field.

Growing research have focused on the porphyrin-based polymer to inhibit the aggregation behavior of porphyrins and enhance the NLO performance in solution (Wang et al., 2022). For example, Qiu et al. used porphyrin as an initiator to prepare Por-PMMA *via* Atom Transfer Radical Polymerization. Due to the steric hindrance of PMMA, the aggregation behavior of porphyrin was effectively inhibited, and its NLO performance was improved in the solvent (Qiu et al., 2013). Du et al. reported the introduction of porphyrin into the main chain of poly (arylene ether sulfone) and a large third order optical susceptibility was obtained (Du et al., 2016). Although the aggregation was effectively reduced, but the decrease of the content of porphyrin in the polymer was decreased with the increase of the molecular weight (MW) of porphyrin-based polymer correspondingly, which has a negative influence on NLO property. Other studies showed that porphyrins and nanomaterials were prepared into nanohybrids, and the NLO performance of the nanohybrids could be improved because of the PET between porphyrins and nanomaterials (Wang et al., 2014; Wang A. et al., 2020; Fu et al., 2022). Consequently, the combination of porphyrin-based polymers and nanomaterials might offer a better solution to further increase the NLO performance of porphyrin-based materials (Wan et al., 2012; Liu et al., 2013; Garg et al., 2017; Liang et al., 2022).

Graphite phase carbon nitride (g-C_3_N_4_) is a semiconducting nanomaterial with a stacked conjugated structure (Ji et al., 2017). Due to the medium bandgap, fast electron-hole separation efficiency and high carrier mobility (Alenizi et al., 2019; Zhang and Liang, 2019; Daraie et al., 2021; Vavilapalli et al., 2021), g-C_3_N_4_ has recently been utilized for constructing NLO materials. Park et al. prepared nanohybrids by combining metal oxide with g-C_3_N_4_, which showed excellent imaginary third-order susceptibility (*Im* [χ^(3)^]) in ethanol (Sridharan et al., 2015). By combining Ag quantum dots with g-C_3_N_4_, Sridharan et al. observed good NLO properties of the nanohybrid due to the energy transfer and electron transfer mechanisms (Sridharan et al., 2014). The electron transfer phenomenon also existed when porphyrin was combined with g-C_3_N_4_. Zhu et al. reported that the remarkable photoinduced electron transfer (PET) process was observed under visible light irradiation by combining porphyrin with g-C_3_N_4_ (Zhu et al., 2020). Thus, combining porphyrin with g-C_3_N_4_ might be a feasible method to improve NLO performance. However, most of the research on the NLO performance of porphyrins has been conducted in liquid systems, which was not conducive to the practical application (Wang A. et al., 2021; Rohal et al., 2022). Moreover, the direct doping of porphyrins and g-C_3_N_4_ in solid matrices would jeopardize NLO performance due to poor dispersion (Qiu et al., 2013). Therefore, it may be feasible to covalently bond g-C_3_N_4_ with porphyrin-based polymers to improve the dispersibility and NLO performance in the solid matrix. As far as we know, there was no literature on the porphyrin-based polymers covalently functionalized g-C_3_N_4_ nanohybrids for NLO research.

In our previous report, the comb-shaped porphyrin-based polymers [PPorx-C≡CH (x = 1, 2 and 3)] had been constructed by Reversible Addition-Fragmentation Chain Transfer Polymerization (Liang et al., 2022). In our work, a flurry of original porphyrin-based polymers covalently functionalized g-C_3_N_4_ (PPorx-g-C_3_N_4_ (x = 1, 2 and 3)] nanohybrids were synthesized *via* azide-alkyne click chemistry. The PPorx-C≡CH with high grafting density and highly flexible main chains could effectively inhibit the aggregation of porphyrin molecules (Chang et al., 2022). In addition, a significant PET process could occur between porphyrin-based polymer [PPorx (x = 1, 2 and 3)] group and g-C_3_N_4_ group in the nanohybrids. With the unique structural features of the nanohybrids, the obtained PPorx-g-C_3_N_4_ exhibited improved NLO performance through the synergistic effect of the minimal aggregation properties caused by PPorx-C≡CH and the PET process between *p*Porx group and g-C_3_N_4_ group. Considering practical use, a series of nanohybrid-doped polymethyl methacrylate (PMMA) composites were constructed through solution casting technology and further research the NLO performance. This research presents a novel design strategy for preparing high-performance NLO materials.

## 2 Experimental

### 2.1 Materials and characterization

Details for materials, synthesis and characterization of g-C_3_N_4_, benzoic acid functionalized g-C_3_N_4_ (g-C_3_N_4_-BA) and PPorx-g-C_3_N_4_/PMMA composites were listed in the Supporting Information. The porphyrin-based polymers PPorx-C≡CH were synthesized according to our previous work (PPor1-C≡CH: *M*_n, GPC_, 2.7 × 10^3^ g mol^−1^; *M*_w, MALS_, 3.5 × 10^3^ g mol^−1^; *M*_w_/*M*_n_ (GPC) = 1.01; PPor2-C≡CH: *M*_n, GPC_, 4.2 × 10^3^ g mol^−1^; *M*_w, MALS_, 5.3 × 10^3^ g mol^−1^; *M*_w_/*M*_n_ (GPC) = 1.06; PPor3-C≡CH: *M*_n, GPC_, 7.0 × 10^3^ g mol^−1^; *M*_w, MALS_, 7.0 × 10^3^ g mol^−1^; *M*_w_/*M*_n_ (GPC) = 1.03) (Liang et al., 2022).

### 2.2 Preparation of azide groups functionalized g-C_3_N_4_ (g-C_3_N_4_-N_3_)

In a typical procedure, g-C_3_N_4_-BA (50 mg) and anhydrous DMF (1 ml) were dispersed in SOCl_2_ (30 ml) and stirred for 24 h in ice-water bath. The mixture was depressurized to remove residual SOCl_2_ to obtain a brown powder. After that, NaN_3_ (185 mg, 30 mmol) in anhydrous DMF (30 ml) was added to the above mixture and stirred in ice-water bath for another 24 h. The obtained mixture was centrifuged, washed alternately with deionized water and absolute ethanol to eliminate the sodium salts and residual DMF and then dried under vacuum over night to obtain g-C_3_N_4_-N_3_ as a yellow powder (52 mg).

### 2.3 Preparation of PPorx-g-C_3_N_4_ (x = 1, 2 and 3)

The PPor1-g-C_3_N_4_ was synthesized *via* copper-catalyzed azide-alkyne click chemistry. g-C_3_N_4_-N_3_ (5 mg) was dispersed in anhydrous DMF (5 ml). PPor1-C≡CH (3 mg, 0.001 mmol), CuBr (0.14 mg, 0.001 mmol) and *N*, *N*, *N*′, *N*″, *N*″-pentamethyldiethylenetriamine (PMDETA, 2 µl, 0.001 mmol) were added into the above mixture. The dispersion was bubbled with N_2_ gas for 30 min and placed in an oil bath at 45°C. After stirring for 8 h, the reaction was precipitated in cold methanol to remove the organic residues. The final product was centrifuged, washed and dried to obtain PPor1-g-C_3_N_4_ as a brown powder (6 mg).

PPor2-g-C_3_N_4_ and PPor3-g-C_3_N_4_ were prepared in a similar way of PPor1-g-C_3_N_4_, with PPor2-C≡CH (3 mg, 0.7 μmol) and PPor3-C≡CH (3 mg, 0.4 μmol), respectively.

## 3 Results and discussion

### 3.1 Synthesis and characterization

**SCHEME 1:**
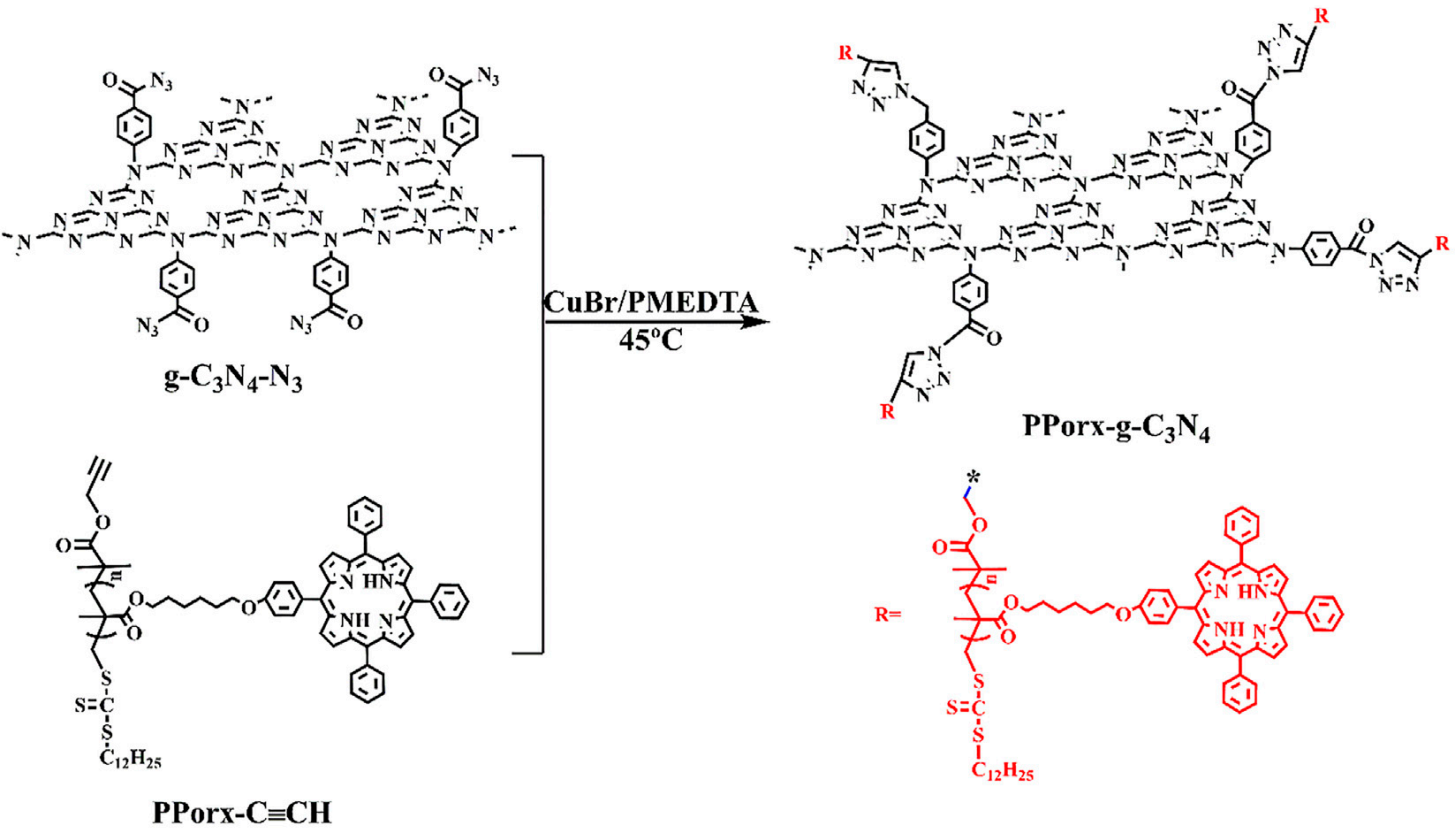
The synthesis route of PPorx-g-C_3_N_4_.

**FIGURE 1:**
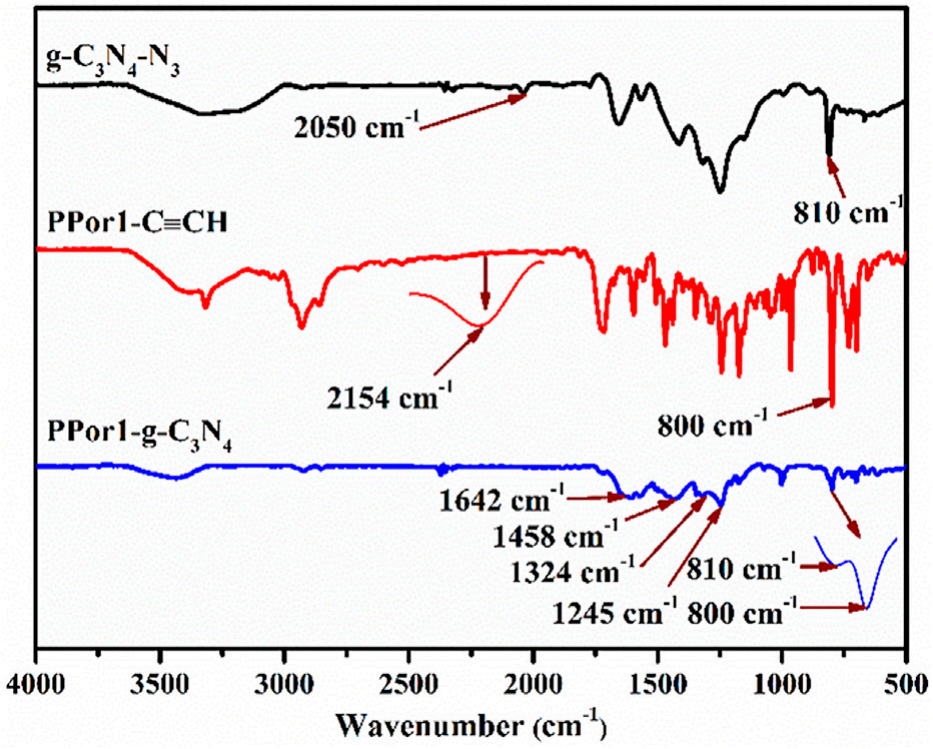
The FT-IR spectra of g-C_3_N_4_-N_3_, PPor1-C≡CH and PPor1-g-C_3_N_4_.

The synthetic process of PPorx-g-C_3_N_4_ nanohybrid was illustrated in Scheme 1. The PPorx-g-C_3_N_4_ nanohybrids were obtained through the click chemistry between PPorx-C≡CH and g-C_3_N_4_-N_3_. Due to their similar structure, PPor1-g-C_3_N_4_ was analyzed to dissect the structure of PPorx-g-C_3_N_4_. Figure 1 shows the Fourier transform infrared (FT-IR) spectra of g-C_3_N_4_-N_3_, PPor1-C≡CH and PPor1-g-C_3_N_4_. The stretching vibration of -C≡CH (2154 cm^−1^) and -N_3_ (2050 cm^−1^) were observed for PPor1-C≡CH and g-C_3_N_4_-N_3_, respectively. For PPor1-g-C_3_N_4_, the -N_3_ and -C≡CH peaks disappeared, the characteristic peaks at 810, 1245, 1324, 1458 and 1642 cm^−1^ from the g-C_3_N_4_ group, and 800 cm^−1^ from porphyrin group were observed, indicating the successful combination of PPor1-C≡CH with g-C_3_N_4_-N_3_.

**FIGURE 2:**
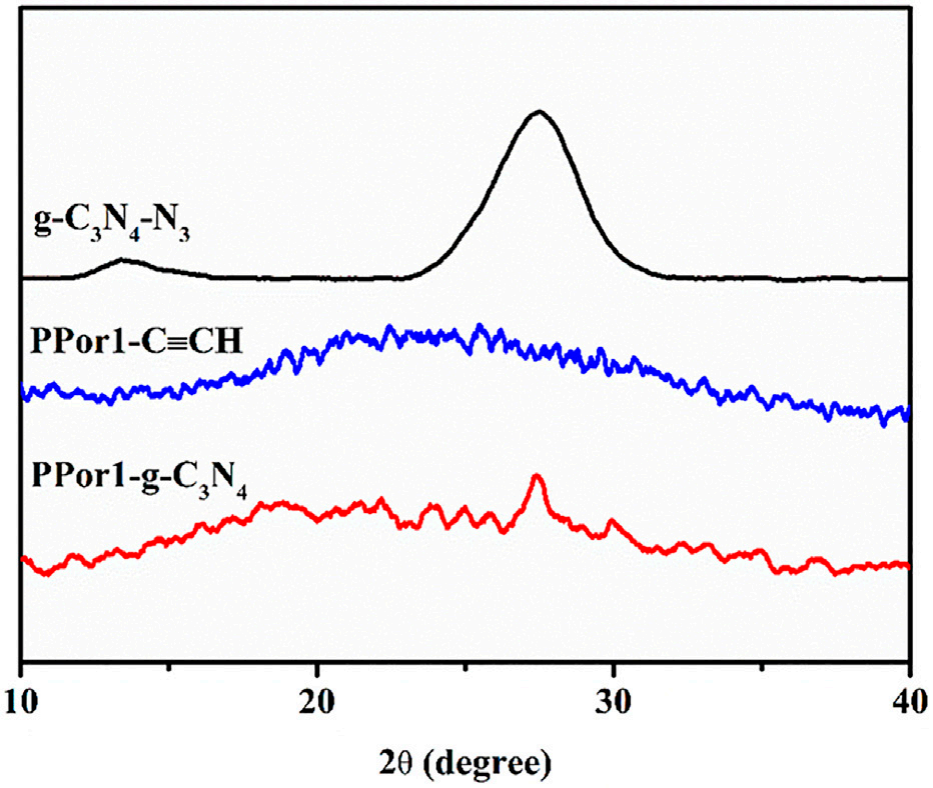
XRD patterns of g-C_3_N_4_-N_3_, PPor1-C≡CH and PPor1-g-C_3_N_4_.

The crystal structure of PPor1-g-C_3_N_4_ was further determined by X-ray diffractometer (XRD). As indicated in Figure 2, a broad peak in the range from 15° to 35° of PPor1-C≡CH was due to its indeterminate structure. The peaks of (100) and (002) at 13.4° and 27.4° of g-C_3_N_4_-N_3_ indicated the interlayer stacking of aromatic rings and the in-plane repeat period in g-C_3_N_4_ (Xu et al., 2021). The amorphous structure of PPor1-g-C_3_N_4_ was due to the distruction of the ordered structure of g-C_3_N_4_-N_3_ by the combination with PPor1-C≡CH.

**FIGURE 3:**
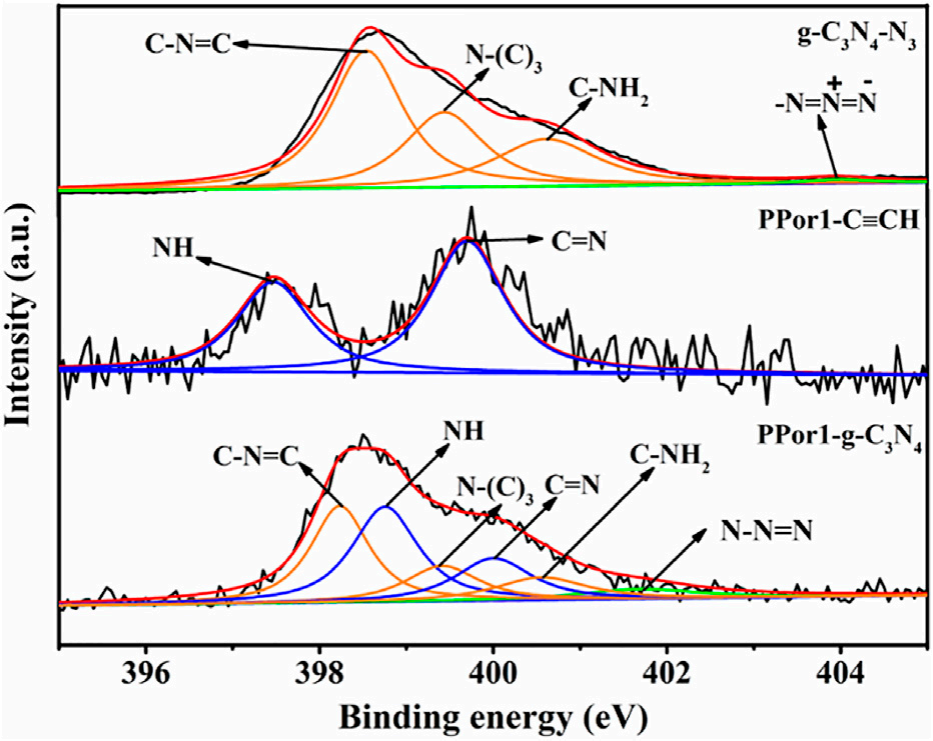
(A) N 1s XPS spectra of g-C_3_N_4_-N_3_, PPor1-C≡CH and PPor1-g-C_3_N_4_.

X-ray photoelectron spectroscopy (XPS) was used to confirm the covalent attachment between PPor1 group and g-C_3_N_4_ group. In the survey spectra (Supplementary Figure S4), PPor1-g-C_3_N_4_ was constructed by C, N, O and S. As shown in Figure 3, the six characteristic peaks in PPor1-g-C_3_N_4_ could be divided into three groups: the characteristic peaks at 398.8 and 400.1 eV belonging to the N of NH and C=N in the pyrrole ring of PPor1 group; the characteristic peaks at 398.3, 399.5 and 400.6 eV belonging to the N of C-N=C, N-(C)_3_ and C-NH_2_ of g-C_3_N_4_, respectively (Xu et al., 2021); the characteristic peak located at 402.0 eV assigned to the N of the triazole ring in PPor1-g-C_3_N_4_(Wipperman et al., 1991; Liu et al., 2016), indicating that PPor1 group was covalently attached to g-C_3_N_4_ group *via* the click reaction. In addition, compared with g-C_3_N_4_-N_3_ and PPor1-C≡CH, the peaks of g-C_3_N_4_ group in PPor1-g-C_3_N_4_ shift towards lower binding energy, while the peaks of porphyrin shift towards higher binding energy, respectively, which could be attributed to the disappearance of the alkynyl group in PPorx-C≡CH and the azide group in g-C_3_N_4_-N_3_ after the click reaction between PPorx-C≡CH and g-C_3_N_4_-N_3_ and the formation of the triazole ring, leading to the change of the chemical environment of N in porphyrin and g-C_3_N_4_ (Li X. et al., 2022; Shi et al., 2022). The results of XPS proved the covalently linking between PPor1 group and g-C_3_N_4_ group.

**FIGURE 4:**
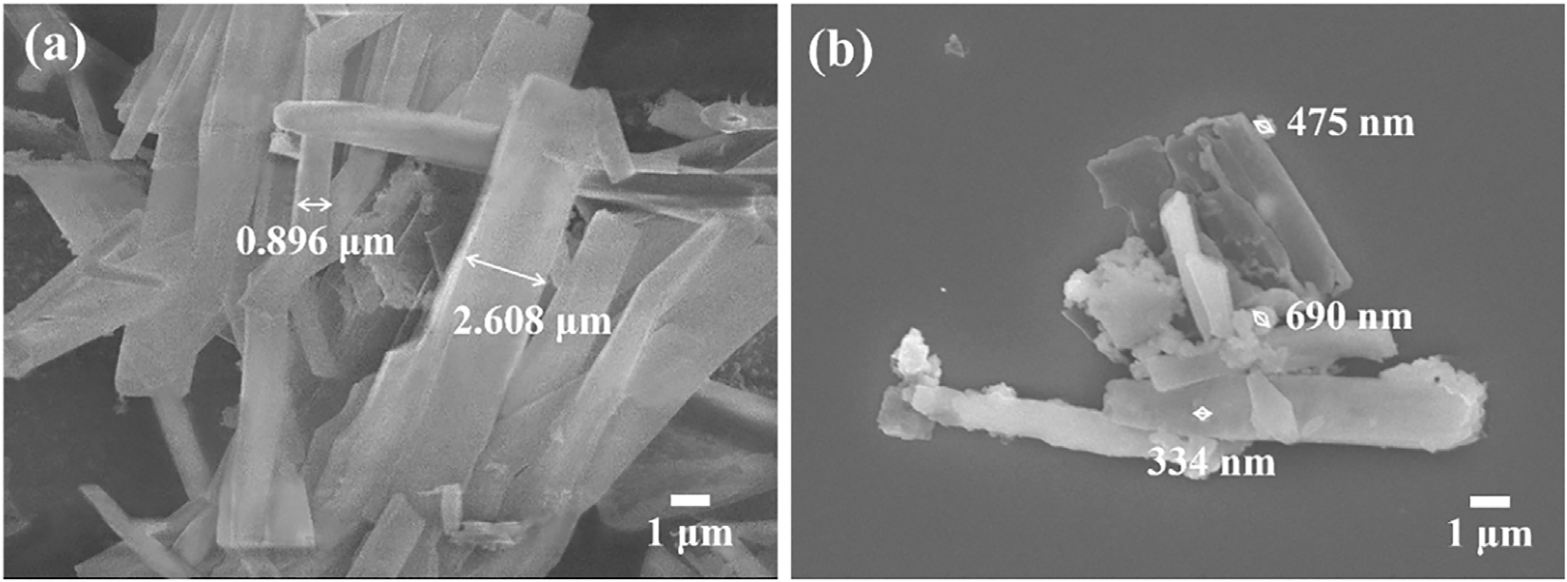
SEM images of **(A)** g-C_3_N_4_-N_3_ and **(B)** PPor1-g-C_3_N_4_.

To further investigate the morphology of material, scanning electron microscope (SEM) was carried out. As shown in Figure 4A, g-C_3_N_4_-N_3_ showed tubular morphology with a smooth surface, and its tube diameters varies from 0.896 μm to 2.608 μm. In Figure 4B, PPor1-g-C_3_N_4_ also exhibited an obvious tubular structure, and there were irregularly shaped particles with a size of several hundred nm on the surface, which might relate to the introduction of *p*Porx group. The FT-IR, XRD, XPS, and SEM together confirmed the successful preparation of PPor1-g-C_3_N_4_.

### 3.2 Optical and physical properties

**FIGURE 5:**
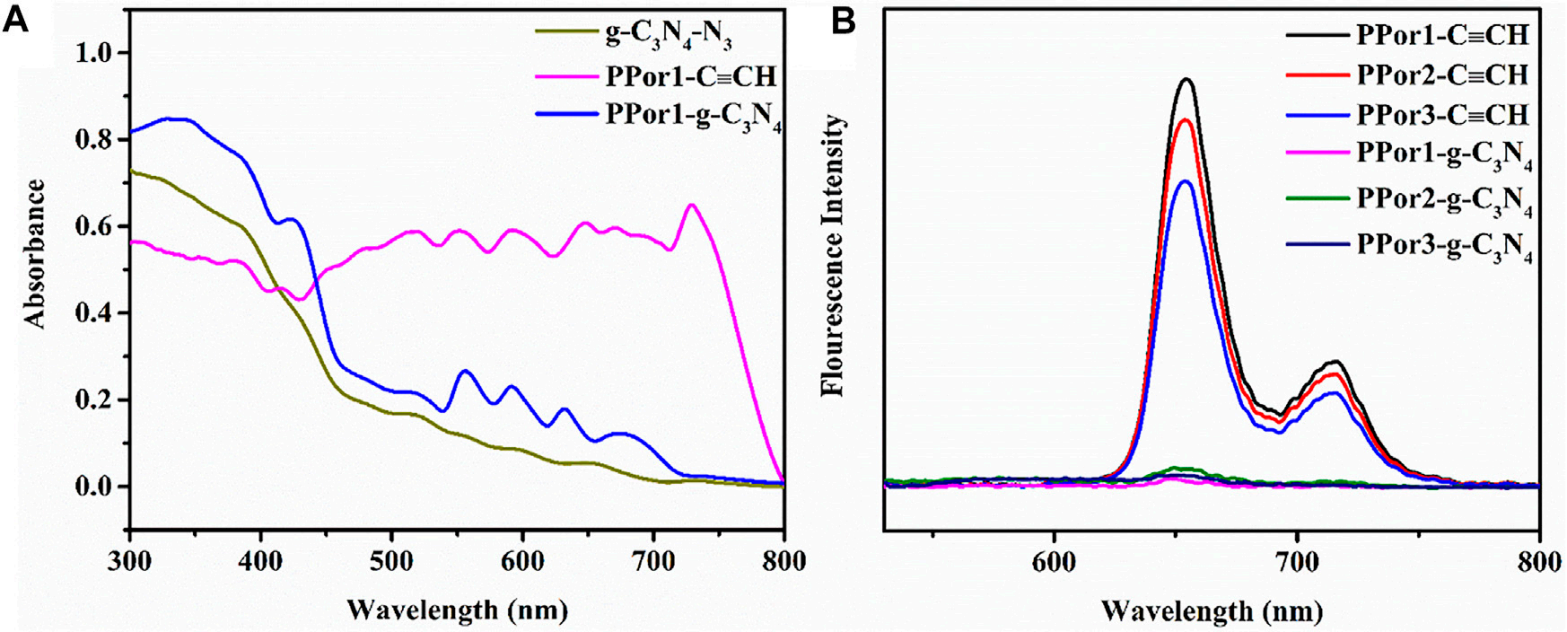
**(A) *DRS of PPor1-C*** ≡CH ***,*** g-C_3_N_4_-N_3_ ***and PPor1-g-C***_***3***_ ***N***_***4***_ ***.* (B)** PL emission spectra of PPor1-C≡CH (6 μmol/ml), PPor2-C≡CH (3.63 μmol/ml), PPor3-C≡CH (2.26 μmol/ml) and PPor1-g-C_3_N_4_∼PPor3-g-C_3_N_4_ (0.05 mg/ml) under 370 nm excitation.

As shown in the UV-vis diffuse reflection spectra (DRS) (Figure 5A), the g-C_3_N_4_-N_3_ exhibited an absorption edge at ca. 450 nm, which is consistent with the literature report (Zhou et al., 2018). A broad peak from 462 to 600 nm derived from the n-π* transition of heptazine ring unit in the g-C_3_N_4_-N_3_ (Xu et al., 2019). PPor1-C≡CH possessed a Soret band at around 417 nm and four weak bands at 500–700 nm attributed to Q bands of porphyrin group. After the click chemistry between g-C_3_N_4_-N_3_ and PPor1-C≡CH, the peaks of porphyrin group in PPor1-g-C_3_N_4_ were red-shifted compared with that in PPor1-C≡CH, which might be involved in the electron interactions between PPor1 group and g-C_3_N_4_ group. From the photoluminescence (PL) spectra (Figure 5B), the three porphyrin-based polymers PPorx-C≡CH exhibited two emission bands at 661 and 725 nm. Besides, the fluorescence emission intensity of PPorx-C≡CH decreases as the degree of polymerization of PPorx-C≡CH increases, which could be explained as follows: as the increase of the degree of polymerization of PPorx-C≡CH, the concentration of porphyrin increases, resulting in a self-quenching phenomenon which is caused by the concentration quenching effect, and this phenomenon becomes more obvious with the increase of the degree of polymerization of PPorx-C≡CH (Grenoble et al., 2005; Loman-Cortes et al., 2021; Li C. et al., 2022). Notably, the PPorx-g-C_3_N_4_ exhibited obvious fluorescence quenching compared with PPorx-C≡CH, and the fluorescence characteristic peak (666 nm) had a redshift of 5 nm, which might be owing to the PET between *p*Porx group and g-C_3_N_4_ group in PPorx-g-C_3_N_4_.

**FIGURE 6:**
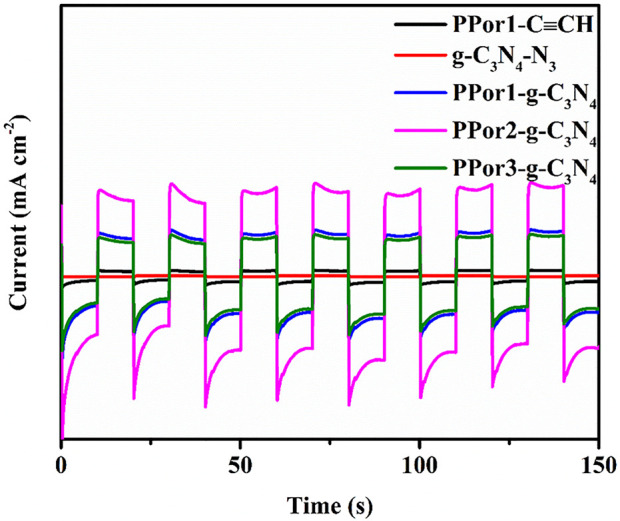
Photocurrent responses of PPor1-C≡CH, g-C_3_N_4_-N_3_ and PPorx-g-C_3_N_4_ (x = 1, 2 and 3) under chopped illumination.

The photocurrent response experiment of PPor1-C≡CH, g-C_3_N_4_-N_3_ and PPorx-g-C_3_N_4_ (x = 1, 2 and 3) were used to further investigate the PET effect between *p*Porx group and g-C_3_N_4_ group in PPorx-g-C_3_N_4_. As shown in Figure 6, the photocurrent response of PPor1-g-C_3_N_4_ was enhanced compared to PPor1-C≡CH and g-C_3_N_4_-N_3_, indicating that the PET existed between PPor1 group and g-C_3_N_4_ group in PPor1-g-C_3_N_4_ under visible light irradiation. In addition, with the increase of MW of *p*Porx group in PPorx-g-C_3_N_4_, the electron transfer effect of PPorx-g-C_3_N_4_ was observed to first increased and then decreased. Among them, PPor2-g-C_3_N_4_ showed the strongest current density, proving that PPor2-g-C_3_N_4_ has the strongest electron transfer effect.

**FIGURE 7:**
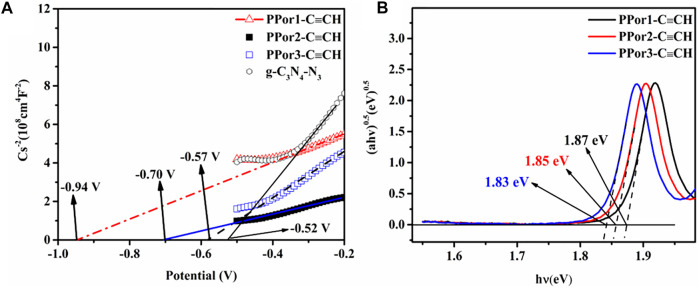
**(A)** Mott-Schottky plots of g-C_3_N_4_-N_3_, PPor1-C≡CH, PPor2-C≡CH and PPor3-C≡CH. **(B)** Tauc plots of PPor1-C≡CH, PPor2-C≡CH and PPor3-C≡CH.

The electrochemical experiments were further carried out to evaluate the effect of the MW of PPor-C≡CH on the electron transfer effect of PPorx-g-C_3_N_4_. As shown in Figure 7A, the Mott-Schottky (MS) plots of g-C_3_N_4_-N_3_, PPor1-C≡CH, PPor2-C≡CH and PPor3-C≡CH exhibited a positive slope, which indicated that all samples were n-type semiconductors (Yang et al., 2020). The flat band potentials (*E*_fb_) of g-C_3_N_4_-N_3_, PPor1-C≡CH, PPor2-C≡CH and PPor3-C≡CH were -0.52 V, -0.94 V, -0.70 V and -0.57 V, respectively, which was measured from the intersection of Cs^−2^–0 linear curve. And the CB edge potential (*E*_CB_) of g-C_3_N_4_-N_3_, PPor1-C≡CH, PPor2-C≡CH and PPor3-C≡CH were calculated to be -0.50 V, -0.92 V, -0.68 V and -0.55 V, respectively, which according to the formula (*E*_CB_ (NHE, pH = 7) = *E*_fb_ (SCE, pH = 7) +0.225–0.2) (Wang et al., 2017). Furthermore, the bandgap energy (*E*_g_) values of g-C_3_N_4_-N_3_ was calculated to be 2.50 eV, PPor1-C≡CH, PPor2-C≡CH, and PPor3-C≡CH were calculated to be 1.87 eV, 1.85 eV and 1.83 eV, respectively, based on the Tauc Plot transformed from UV-vis DRS spectra (Supplementary Figure S5 and Figure 7B) (Xu et al., 2019). The reduction of the bandgap of PPorx-C≡CH is associated with the increased conjugacy of porphyrin, which becomes more pronounced as the MW of PPorx-C≡CH increases (Qiu et al., 2013). Based on the empirical formula *E*_VB_ = *E*_CB_ + *E*_g_, the VB edge potential (*E*_VB_) of g-C_3_N_4_-N_3_, PPor1-C≡CH, PPor2-C≡CH and PPor3-C≡CH were calculated to be 2.00 V, 0.95 V, 1.17 V and 1.28 V, respectively. Consequently, the interlaced band structures of g-C_3_N_4_-N_3_ and PPorx-C≡CH could be obtained (Ismael, 2022).

**FIGURE 8:**
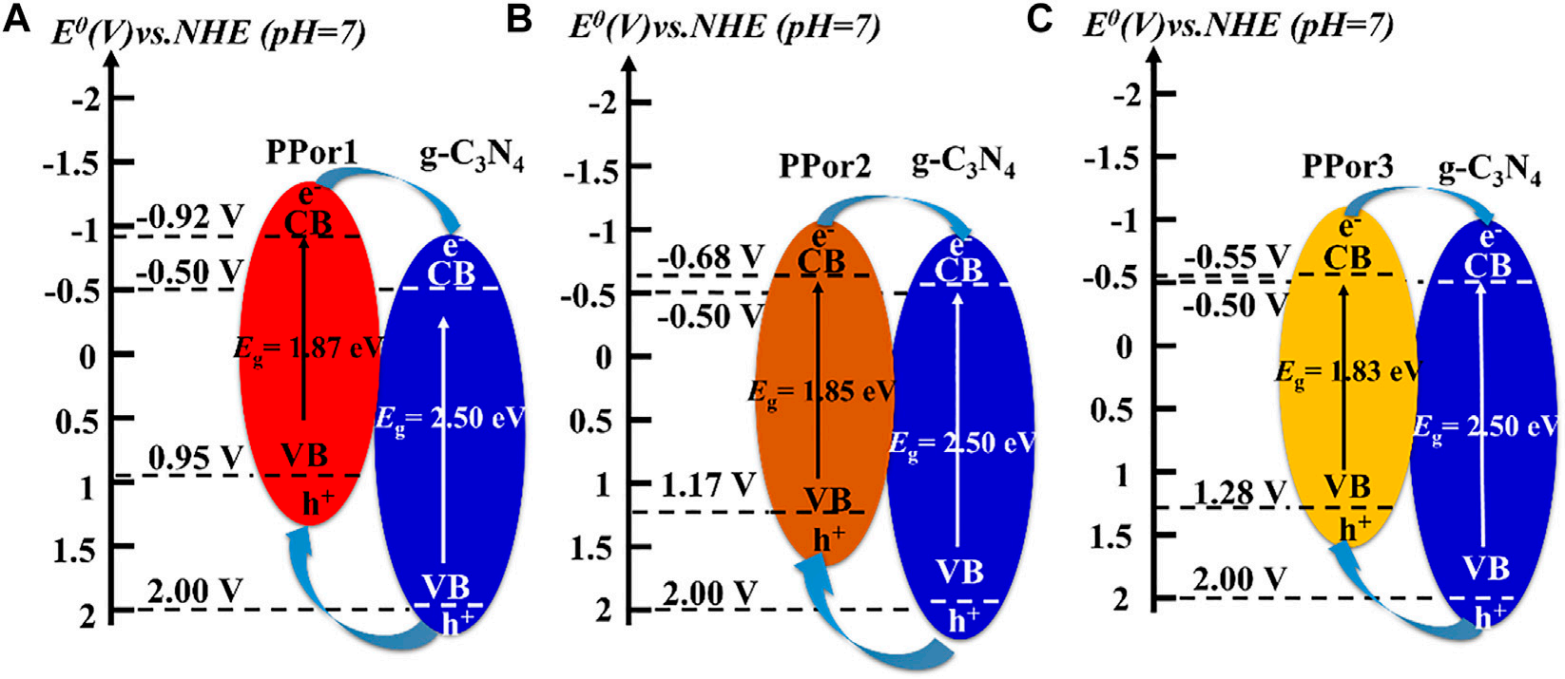
The schematic diagrams of the proposed electron transfer under visible light irradiation in **(A)** PPor1-g-C_3_N_4_, **(B)** PPor2-g-C_3_N_4_ and **(C)** PPor3-g-C_3_N_4_.

The electron transfer process in the PPorx-g-C_3_N_4_ is shown in Figure 8. Under irradiation, *p*Porx group and g-C_3_N_4_ group could be excited and produce abundant e^−^ and h^+^ at the same time. Due to their staggered band structure, the electron transfer process was as follows: e^−^ could transfer from the CB of PPorx group to the CB of g-C_3_N_4_ group and h^+^ on the VB of g-C_3_N_4_ group could move to the VB of PPorx group. Thus, PPorx group could behave as the electron donor, and g-C_3_N_4_ group as the electron acceptor in PPorx-g-C_3_N_4_ nanohybrids. Moreover, as the MW of PPorx-C≡CH increased, the energy difference between the *E*_CB_ of PPorx-C≡CH and the *E*_CB_ of g-C_3_N_4_-N_3_ decreased, which might result in lower electron transfer effect. However, the electron transfer efficiency of PPorx-g-C_3_N_4_ was observed to increase first and then decrease based on the photocurrent response experiment, which might be explained as follows. The PPorx group in the PPorx-g-C_3_N_4_ was increased after the click chemistry reaction with the increase of the MW of PPorx-C≡CH, causing the enhanced electron transfer effect. However, the MW of PPorx-C≡CH is gradually increased to a certain extent, and it contributes to steric hindrance increase, which hinders the click chemistry reaction between PPorx-C≡CH and g-C_3_N_4_-N_3_, and then, the *p*Porx group in the PPorx-g-C_3_N_4_ was decreased, leading to a diminished electron transfer effect. Consequently, the electron transfer effect exerts a trend of first increasing and then decreasing.

### 3.3 Non-linear optical properties of PPorx-g-C_3_N_4_ nanohybrids

**FIGURE 9:**
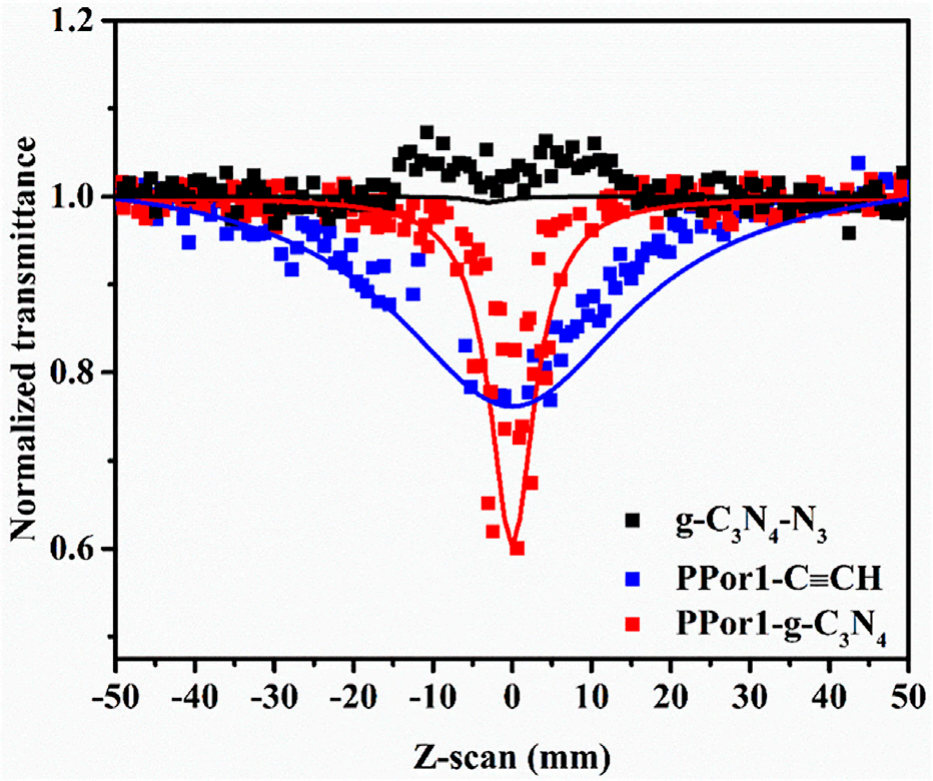
The open aperture Z-scan tests of g-C_3_N_4_-N_3_, PPor1-C≡CH and PPor1-g-C_3_N_4_ in MMA (0.05 mg/ml).

The NLO performances of PPorx-g-C_3_N_4_ nanohybrids were investigated in MMA by the Z-scan technique with 7 ns laser pulses of 532 nm. Generally, the value of the non-linear absorption coefficient (*β*_eff_) was used to evaluate the reverse saturable absorption (RSA) performance. The excellent RSA performance would result in a large *β*_eff_ and a deep “V" shaped absorption curve. As shown in Figure 9, the RSA performance of PPor1-g-C_3_N_4_ nanohybrid was better than the corresponding PPor1-C≡CH and g-C_3_N_4_-N_3_. The *β*_eff_ of PPor1-g-C_3_N_4_ was calculated to be 3.4 × 10^–9^ m/W, and *Im* [χ^(3)^] value was calculated to be 1.11 × 10^–10^ esu, which was ca. 5.76 times higher if compared to PPor1-C≡CH (*Im* [χ^(3)^] of 0.16 × 10^–10^ esu), attributing to the PET behavior between PPor1 group and g-C_3_N_4_ group in PPor1-g-C_3_N_4_. The NLO properties of PPorx-g-C_3_N_4_ in MMA are listed in Table 1 [for comparison, the results of covalently linked 5-(4-hydroxylphenyl)-10,15,20-triphenylporphyrin-g-C_3_N_4_ (Por-g-C_3_N_4_) is also provided (Supplementary Figure S6)]. From Table 1, PPor1-g-C_3_N_4_ and PPor2-g-C_3_N_4_ show larger *Im* [χ^(3)^] value than that of Por-g-C_3_N_4_, proving that the introduction of porphyrin-based polymer into g-C_3_N_4_ effectively improve the aggregation behavior of porphyrins and enhanced the NLO performance of the nanohybrids. PPor2-g-C_3_N_4_ exhibited the best NLO performance among PPorx-g-C_3_N_4_ with *β*_eff_ of 4.5 × 10^–9^ m/W and *Im* [χ^(3)^] of 1.47 × 10^–10^ esu, respectively, due to the efficient PET from PPor2 group to g-C_3_N_4_ group in PPor2-g-C_3_N_4_, so that PPor2-g-C_3_N_4_ exhibits the best NLO performance in MMA.

**TABLE 1:** NLO properties of as prepared samples in MMA.

Sample^ab^	Linear transmittance (%)	*β*_eff_ (m/W)	*Im* [χ^(3)^] (esu)
PPor1-C≡CH	65	0.5 × 10^–9^	0.16 × 10^–10^
g-C_3_N_4_-N_3_	70	0.1 × 10^–10^	0.03 × 10^–10^
Por-g-C_3_N_4_	70	0.8 × 10^–9^	0.27 × 10^–10^
PPor1-g-C_3_N_4_	68	3.4 × 10^–9^	1.11 × 10^–10^
PPor2-g-C_3_N_4_	65	4.5 × 10^–9^	1.47 × 10^–10^
PPor3-g-C_3_N_4_	69	0.7 × 10^–9^	0.22 × 10^–10^

^a^ Peak intensity for each independent Z-scan measurement was -13 μJ. The excitation source was 7 ns laser pulses of 532 nm wavelength.

^b^ c = 0.05 mg/ml.

**FIGURE 10:**
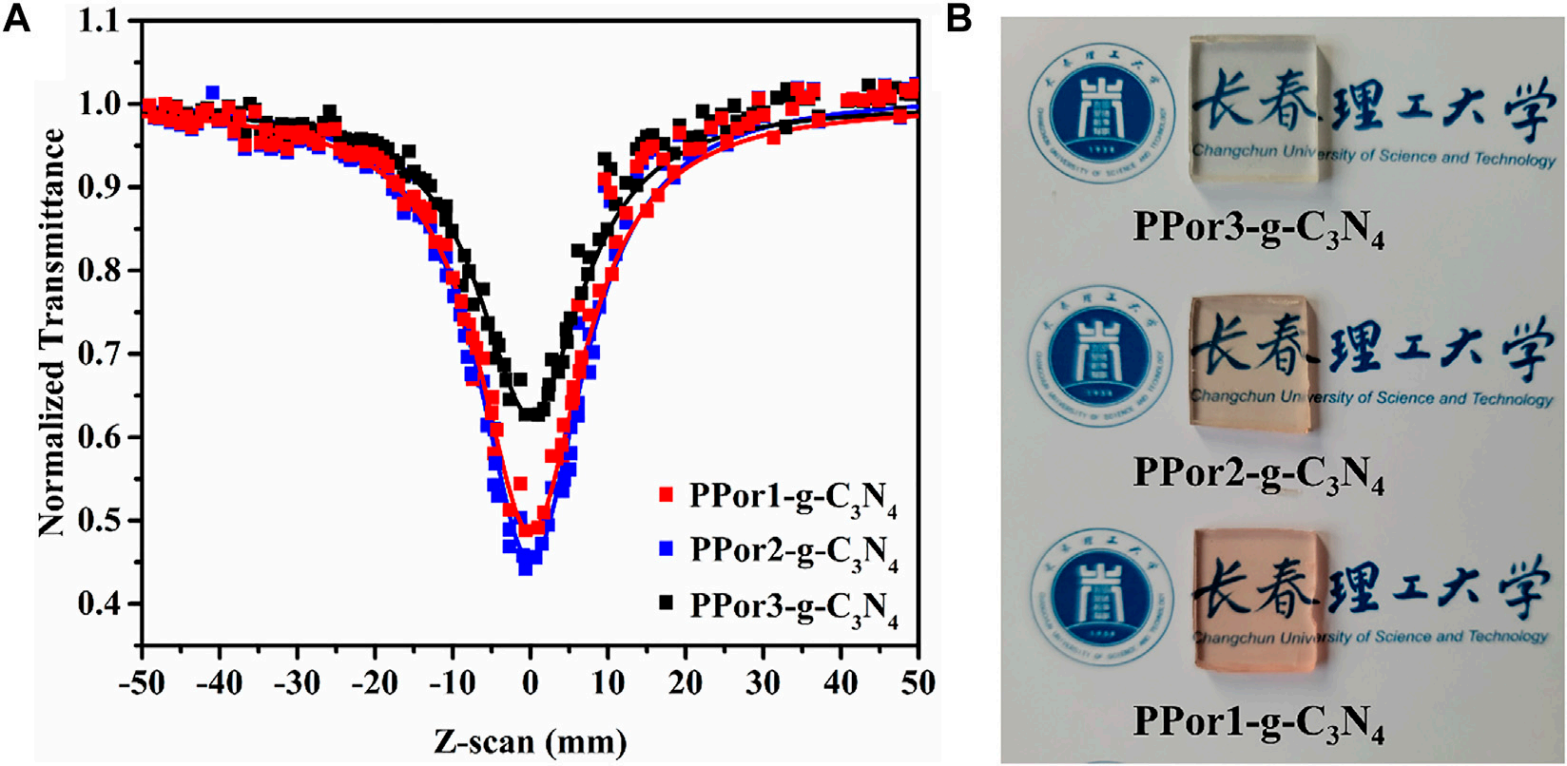
**(A)** The open aperture Z-scan tests of PPorx-g-C_3_N_4_/PMMA composites with the doping concentration of 0.05 mg/ml. **(B)** Photographs of PPorx-g-C_3_N_4_/PMMA composites with 0.05 mg/ml doping concentrations.

To study the practicality of PPorx-g-C_3_N_4_, the PPorx-g-C_3_N_4_ was doped PMMA to form PPorx-g-C_3_N_4_/PMMA composites *via* solution casting technology (described in supporting information). Figure 10A showed the RSA performance of PPorx-g-C_3_N_4_/PMMA (0.05 mg/ml), and the results were listed in Table 2. Compared with PPorx-g-C_3_N_4_ in MMA (Table 1), the NLO performance of PPorx-g-C_3_N_4_/PMMA composites was improved, which might be owing to the weaker aggregation effect in the solid matrix (Wang J. et al., 2020). Among them, PPor2-g-C_3_N_4_/PMMA composite exhibited the deepest trough, with the excellent *β*_eff_ of 7.2 × 10^–9^ and *Im* [χ^(3)^] of 2.36 × 10^–10^ esu, respectively. Furthermore, the photographs of PPor1-g-C_3_N_4_/PMMA, PPor2-g-C_3_N_4_/PMMA and PPor3-g-C_3_N_4_/PMMA composites were shown in Figure 10B, all of them showed excellent transparency, demonstrating the great potential in practical application.

**TABLE 2:** NLO properties of as prepared samples in PMMA.

Sample^ab^	Linear transmittance (%)	*β*_eff_ (m/W)	*Im* [χ^(3)^] (esu)
PPor1-g-C_3_N_4_/PMMA	65	4.8 × 10^–9^	1.58 × 10^–10^
PPor2-g-C_3_N_4_/PMMA	67	7.2 × 10^–9^	2.36 × 10^–10^
PPor3-g-C_3_N_4_/PMMA	63	2.8 × 10^–9^	0.92 × 10^–10^

^a^ Peak intensity for each independent Z-scan measurement was 13 μJ. The excitation source was 7 ns laser pulses of 532 nm wavelength.

^b^ c = 0.05 mg/ml.

**FIGURE 11:**
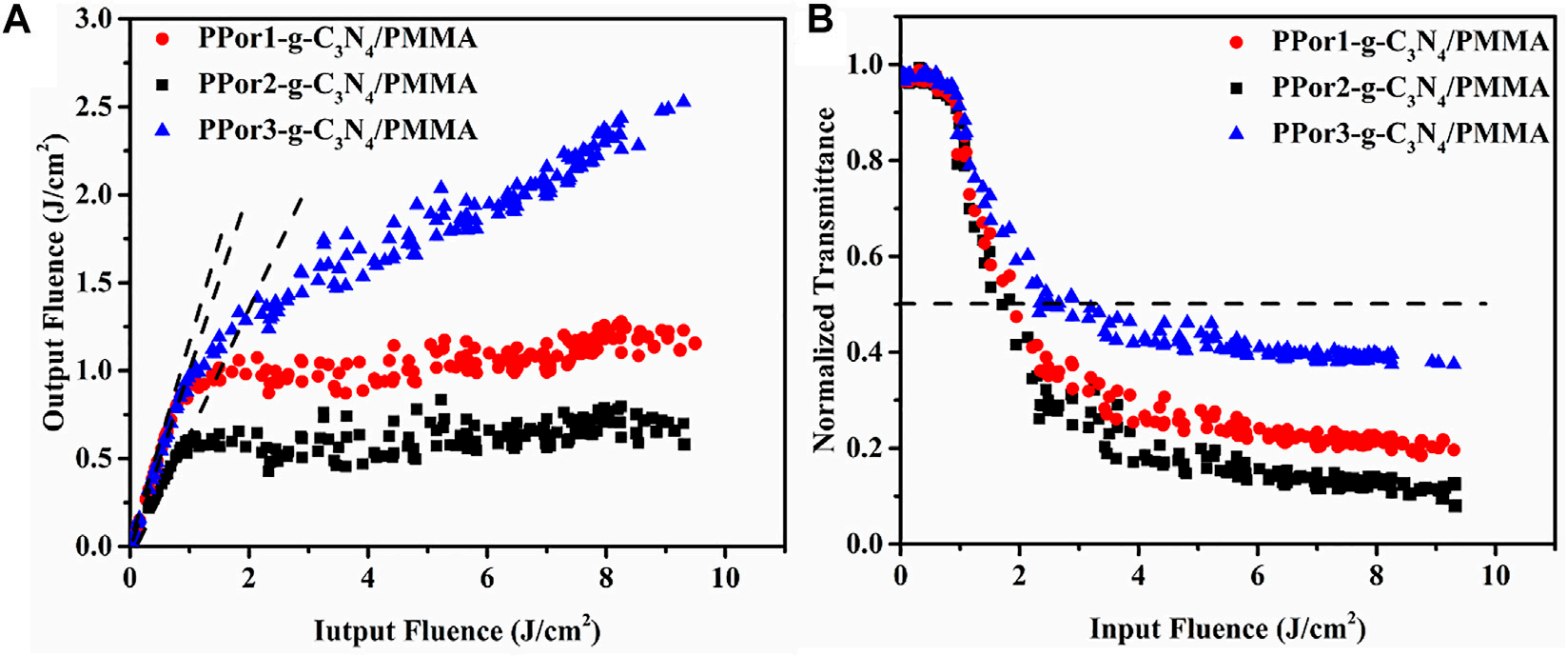
**(A)** The OL performance and **(B)** the non-linear transmittance of PPorx-g-C_3_N_4_/PMMA composites with the doping concentration of 0.05 mg/ml.

The optical limiting (OL) properties of the PPorx-g-C_3_N_4_/PMMA composites are shown in Figure 11A. Under the condition of low input fluence, the output fluence of PPorx-g-C_3_N_4_/PMMA composites promoted with the increase of input fluence, displaying a linear optical property. However, as the input fluence was further increased, PPor1-g-C_3_N_4_/PMMA, PPor2-g-C_3_N_4_/PMMA and PPor3-g-C_3_N_4_/PMMA composites showed obvious non-linear trends, and their initial thresholds were determined to be 0.401 J/cm^2^, 0.058 J/cm^2^ and 0.464 J/cm^2^, respectively. Figure 11B shows the relationship between the input fluence and the normalized transmittance, where the black dotted line represents 50% of the initial transmittance. From Figure 11B, the decreasing normalized transmittance of all PPorx-g-C_3_N_4_/PMMA composites was associated with the increase of the input fluence. Among them, the PPor2-g-C_3_N_4_/PMMA composite exerted the best OL performance, with the limiting threshold of 1.71 J/cm^2^, the minimum transmittance of 8% and the dynamic range of 1.09, respectively, which might be owing to the excellent PET from PPor2 group to g-C_3_N_4_ group in PPor2-g-C_3_N_4_. Some reported OL performances of the porphyrin-based materials are summarized in Table 3, and our OL data demonstrate that PPorx-g-C_3_N_4_/PMMA composites are among the best performing materials for this purpose. In practical application, the damage threshold was an important criterion to measure the stability of the material, interpreting no optical damage occurrence under this input fluence condition. There was no obvious damage observed for PPorx-g-C_3_N_4_/PMMA composites even if the input fluence reached 16 J/cm^2^, which could be owing to the good thermal stability of each fraction in PPorx-g-C_3_N_4_/PMMA composites. These results proved that the PPorx-g-C_3_N_4_/PMMA composites had good application prospects in the OL field.

**TABLE 3:** Summary of the OL parameters of PPorx-g-C_3_N_4_/PMMA and reported works.

Sample	Initial threshold (J/cm^2^)	The dynamic range	References
PPor1-g-C3N4/PMMA	0.401	0.43	this work
PPor2-g-C3N4/PMMA	0.058	1.09	this work
PPor3-g-C3N4/PMMA	0.464	0.70	this work
Pm@HPA in DMF	0.531	0.60	Hassan et al. (2018)
1-GO in DMF	−	0.45	Garg et al. (2017)
Penta (ZnP)C_60_ in DMF	−	0.66	Kulyk et al. (2020)
SWCNT-TPP1 in DMF	−	0.92	Wang et al. (2019)
LaPc in DMF	−	0.27	Ou et al. (2021)

## 4 Conclusion

In summary, a flurry of novel porphyrin-based polymers functionalized g-C_3_N_4_ nanohybrids PPorx-g-C_3_N_4_ had been prepared. The PPorx-g-C_3_N_4_ nanohybrids exhibited improved NLO performance compared to single g-C_3_N_4_-N_3_ and PPorx-C≡CH using the Z-scan technique under 532 nm in ns regimes. Among them, due to the suitable molecular weight and steric hindrance, the efficient PET from PPor2 group to g-C_3_N_4_ group in PPor2-g-C_3_N_4_ gave PPor2-g-C_3_N_4_ the best NLO performance among PPorx-g-C_3_N_4_ with *β*_eff_ of 1.47 × 10^–10^ esu. For practical application, the PPorx-g-C_3_N_4_ doped PMMA composites were prepared by the solution casting method. PPor2-g-C_3_N_4_/PMMA composite exhibited the best *Im* [χ^(3)^] of 2.36 × 10^–10^ esu, initial threshold of 0.058 J/cm^2^ and dynamic range of 1.09, indicating its great potential for practice. This research provided a new strategy for the design of porphyrin-based nanohybrid for NLO application.

## Data Availability

The original contributions presented in the study are included in the article/Supplementary Material, further inquiries can be directed to the corresponding authors.
