# Supplementary material for: Enhanced non-linear optical properties of porphyrin-based polymers covalently functionalized with graphite phase carbon nitride
Source: Front Chem. 2022 Dec 15;10:1102666. doi: 10.3389/fchem.2022.1102666 (PMC9797598; doi:10.3389/fchem.2022.1102666)
Supplement: Supplementary file 1 [file DataSheet1.docx]

**Supporting Information**

**Enhanced nonlinear optical properties of porphyrin-based polymers covalently functionalized with graphite phase carbon nitride**

Chen Liang [^a^](#OLE_LINK1), Xu Cui ^a^, Wenyue Dong [^a^](#OLE_LINK1), Jieming Qin [^a^](#OLE_LINK1)^,^ *, Qian Duan [^a^](#OLE_LINK1)^,^ [^b^](#OLE_LINK2)^,^ *

*a School of Materials Science and Engineering, Changchun University of Science and Technology, Changchun, 130022, China*

*b Engineering Research Center of Optoelectronic Functional Materials, Ministry of Education, Changchun, 130022, China*

**Corresponding author. School of Materials Science and Engineering, Changchun University of Science and Technology, Changchun, 130022, China.*

*E-mail: [duanqian88@hotmail.com](mailto:duanqian88@hotmail.com)*

**Section 1. Materials and Synthesis**

**Materials**

Potassium hydroxide, nitric acid and sodium azide were purchased from Shanghai Macklin Biochemical Co., Ltd. Benzaldehyde, methacryloyl chloride and propargyl bromide were purchased from Aladdin Reagent Company. Methyl methacrylate (MMA) purchased from Shanghai Macklin Biochemical Co., Ltd was distilled at reduced pressure. All other chemicals were purchased from Sinopharm Chemical Reagent Co. and used as received.

**Characterization**

Fourier transform infrared (FT-IR) spectra of the samples were performed on a PerkinElmer Frontier MIR/FIR + SP10 STD spectrometer in KBr plates with a resolution of 4 cm^-1^ scannings from 500-4000 cm^-1^. UV-vis diffuse reflection spectra (DRS) of the samples were measured by Shimadzu UV-3600 spectrophotometer in the range of 200–800 nm. Photoluminescence (PL) spectra were measured on a PerkinElmer LS-55 Fluorescence spectrometer using appropriate filters. The porphyrin-based polymers and nanohybrids fluorescence on excitation at 370 nm was recorded for emission from 530 nm to 800 nm. The slit widths for both the excitation and the emission monochromators were set at 10 nm, the scan speed at 540 nm/min and the resolution at 5 nm. The morphology and microstructure of samples were performed using scanning electron microscopy (SEM) by JEM-6701F (JEOL Ltd.). Transmission electron microscopy (TEM) investigation was performed by Tecnai F20. X-ray photoelectron spectroscopy (XPS) measurements were conducted on an RBD upgraded PHI-5000C ESCA electron spectrometer (PerkinElmer) with an Mg Kα excitation source at 280 eV. X-ray diffraction (XRD) patterns were recorded using a RigakuUltima VI diffractometer, equipped with a 3 kW generator, using Cu Kα radiation (λ = 0.15418 nm) X-ray tube and a Lynx Eye Detector. Profiles were obtained between 5° and 40°, and a count of 60 s was performed at room temperature every 15°. NLO properties were measured by Z-scan measuring system of Suzhou micro nano laser photon technology Co., Ltd. All Z-scan tests were performed using a frequency doubled Nd: Y AG laser with the second harmonic of a Q-switched Nd: YAG laser (1064 nm, 7 ns), which was used as the laser source with a repetition rate of 10 Hz. The liquid samples were placed in a cuvette (internal dimensions: 2 mm×10 mm×5.5 mm, 0.7 mL).

**Electrochemical measurements**

Photocurrent response and Mott Schottky were measured by an electrochemical workstation (CHI660D) using a three-electrode system with Na_2_SO_4_ (0.5 M) solution as the electrolyte. The three-electrode system consisted of a platinum wire electrode, a saturated silver/silver chloride electrode and a working electrode. The working electrode was prepared as follows: 2 mg of sample powder was dispersed in 1.0 mL of absolute ethanol by ultrasound. The suspension was coated on the surface of indium tin oxide (ITO) glass, dried at room temperature and then heated at 65 ℃ for 24 h. A 300 W xenon lamp (CEL-HXF300) with a 400 nm cut-off filter was used as the visible-light source.

**Nonlinear optical parameters**

The measured quantity in open-aperture z-scan experiments of copolymers were the normalized transmittance, given by eqn (1) for a Gaussian pulse.

$T(z)=\frac{1}{\sqrt{\pi}q_{0}(z)}\int_{-\infty}^{\infty} \ln\left[1+q_{0}(z)\text{e}^{\text{-}\tau^{2}}\right]\text{ d}\tau$ (1)

where

$q_{0}(z)=\beta_{\text{eff}}I_{00}L_{\text{eff}}/(1+z^{2}/z_{0}^{2})$ (2)

The nonlinear absorption coefficient (*β*_eff_) could be obtained from the equation. *I*_00_ is the intensity of the beam at the focus. *z* and *z*_0_ are the sample position with respect to the input intensity and the Rayleigh length, respectively. *L*_eff_ is the effective thickness of the sample and was given by using eqn (3).

$L_{\text{eff}}=\frac{1-\text{e}^{-\alpha L}}{\alpha}$ (3)

where α is the linear absorption coefficient and L is the thickness of the sample.

The imaginary component of the third order optical susceptibility (Im [χ^(3)^]) is directly proportional to *β*_eff_ *via* eqn (4).

$\mathrm{Im}\left[\chi^{\left(3\right)}\right]=\frac{n^{2}\varepsilon_{0}c\lambda\beta_{\text{eff}}}{2\text{π}}$ (4)

where *c* and *n* are the speeds of the light in vacuum and the linear refractive index, respectively. *ε*_0_ is the permittivity of free space and *λ* is the wavelength of the laser light.

**Synthesis of 5-(4-hydroxylphenyl)-10,15,20-triphenylporphyrin (Por)**


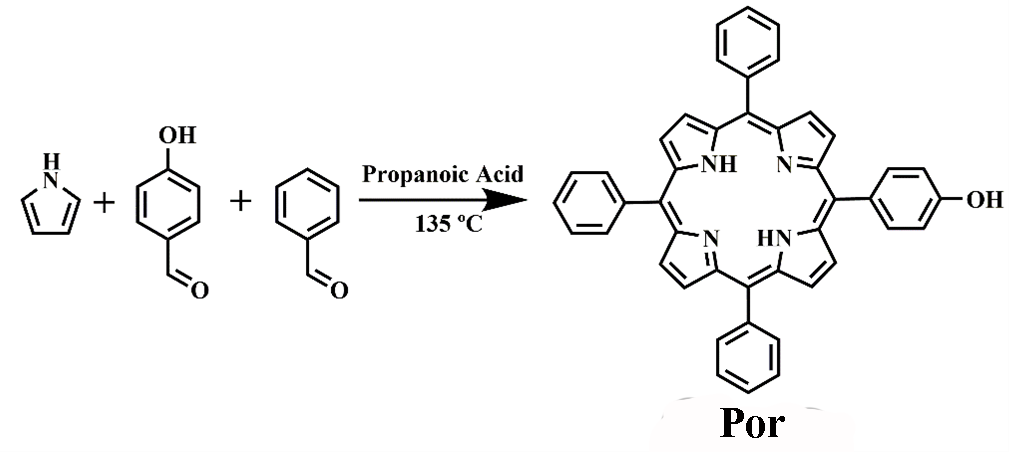


**Scheme S1** The synthesis of **Por**.

It was synthesized according to the literature with modification.(D'Souza *et al.*, 2001) To a solution of benzaldehyde (4.75 mL, 54 mmol) and p-hydroxybenzaldehyde (2.20 g, 18 mmol) in propionic acid (30 mL)，pyrrole (5 mL, 72 mmol) was dropwise added and the mixture was stirred at 135 ºC for 3 h. After cooling to room temperature, the reaction mixture was filtered under vacuum and washed thoroughly with ethanol until the filtrate became clear to obtain purple precipitation. The crude product was purified on a silica gel column with dichloromethane (DCM) as the eluent to afford the **Por** as purple solid. Yield: 0.6 g (10%). FT-IR (KBr pellet) ν: 3500, 3319, 1595, 1487, 1453, 1088, 964, 800, 725 cm^-1^; ^1^H NMR (500 MHz, CDCl_3_), δ ppm: 8.86 (m, 8H), 8.21 (m, 6H), 8.08 (m, 2H), 7.75 (m, 9H), 7.22 (m, 2H), -2.77 (s, 2H).

**
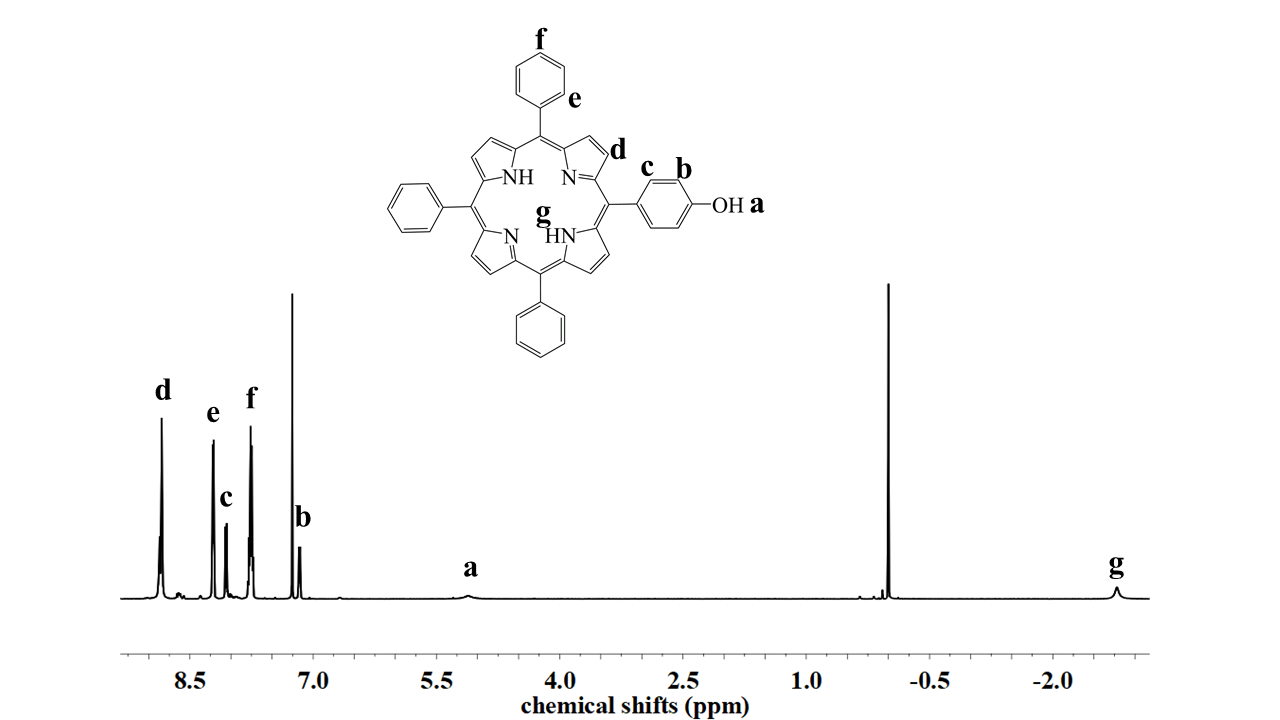
**

**Fig. S1** ^1^H NMR spectrum of **Por** in CDCl_3_.

**Synthesis of graphitic carbon nitride (g-C_3_N_4_)**

It was synthesized according to the literature with modification (Gao *et al.*, 2012). In a typical synthesis procedure, melamine was dissolved in ethylene glycol to make a saturated solution at ambient temperature. nitric acid aqueous solution (60 mL, 0.12 M) was added into the above solution (20 mL) by stirring. The generated white precipitation was collected and washed by ethanol for three times to remove residual nitric acid and ethylene glycol. Then the product was dried at 60 °C for 5 h and sintered at 350 °C for 1 h in air at a heating rate of 10 °C min^-1^ to obtain g-C_3_N_4_ as a yellow powder (237 mg).

**Synthesis of benzoic acid functionalization of g-C_3_N_4_ (g-C_3_N_4_-BA)**

It was synthesized according to the literature with modification (Liao *et al.*, 2020). Firstly, the phenyl carboxylic diazonium salt was fabricated according to ref (Jahan *et al.*, 2010). NaOH (0.28 g) and 4-aminobenzoic acid (0.96 g) were completely dissolved in water (80 mL). Then, NaNO_2_ (0.526 g) was slowly added to the above solution at 0 ºC. The mixture was added to HCl solution (50 mL, 6.5 mol L^-1^) and stirred for 45 min. Afterward, the prepared g-C_3_N_4_ (50 mg) was added to above diazonium salt solution, and stirred for 4 h at 0 °C. Then, the resulted solution was filtered, washed successively with distilled water, ethanol, DMF, and dried at 60 °C overnight to obtain g-C_3_N_4_-BA as an orange powder (87 mg).


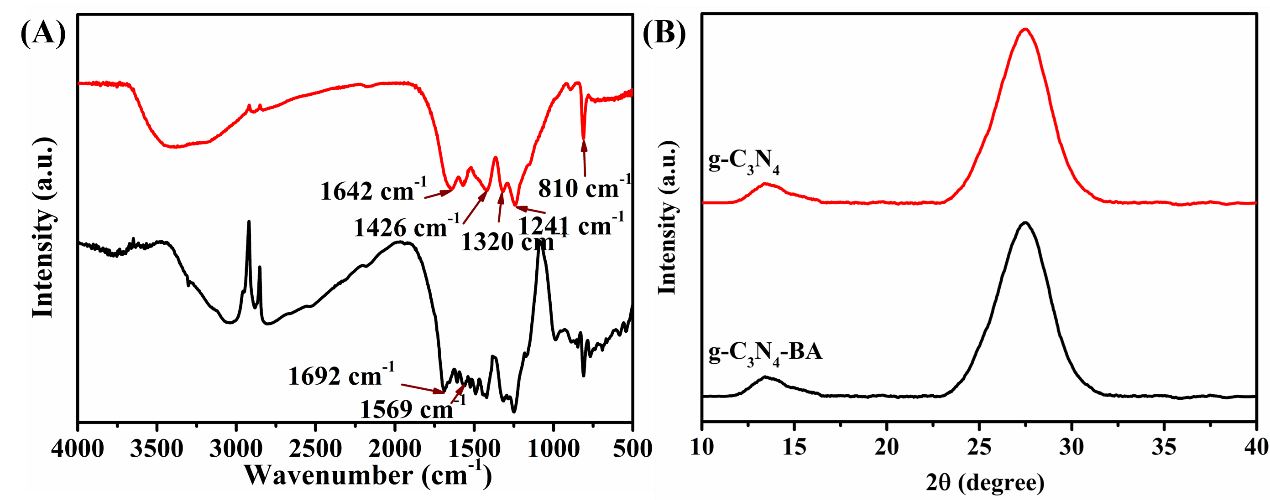


**Fig. S2.** (A) FT-IR and (B) XRD spectra of **g-C_3_N_4_** and **g-C_3_N_4_-BA**.

**Synthesis of 5-(4-hydroxylphenyl)-10,15,20-triphenylporphyrin-g-C_3_N_4_ (Por-g-C_3_N_4_)**

**

**

**Scheme S2** The synthesis of **Por-g-C_3_N_4_**.

Por-g-C_3_N_4_ was prepared by esterification of Por and g-C_3_N_4_-BA. To a solution of Por (3 mg, 4 mmol), 4-Dimethylaminopyridine (0.5 mg, 4 mmol, DMAP) and N, N’-Dicyclohexylcarbodiimide (0.98 mg, 4 mmol, DCC) in anhydrous CH_2_Cl_2_ (20 mL) in an ice bath (0 °C), g-C_3_N_4_-BA (5 mg) was added, and the mixture was stirred at room temperature overnight. The reaction was precipitated in cold methanol to remove the organic residues. The final product was centrifuged, washed and dried to obtain Por-g-C_3_N_4_ as a brown powder (5 mg).





**Fig. S3** FT-IR spectra of **Por-g-C_3_N_4_**, **Por** and **g-C_3_N_4_-BA**.

**Preparation of PPor2-g-C_3_N_4_/PMMA composites**

The preparation method of compositive material PPor2-g-C_3_N_4_/PMMA (0.05 mg/mL) was as followed. The azodiisobutyronitrile (30 mg, 0.18 mmol) as free radical initiator was dissolved in MMA (9.44 g, 94.3 mmol). Then the mixture was stirred at 65 °C until the pre-polymerized solution reaches appropriate viscosity. The PPor2-g-C_3_N_4_ (0.5 mg) was dispersed into DMF (0.2 mL) and added to the pre-polymerized solution until mixed well. The evenly mixed viscous solution was added into mold and heated in an oven at 70 °C for 3 h, 80 °C for 2 h, 90 °C for 1 h and then cooled down to room temperature. After removing the mold, the solid PPor2-g-C_3_N_4_/PMMA composite of 2 mm-thick was obtained. All the other PMMA composites were prepared in the same way for controlled experiments in Z-scan test.

**Section 2.** **XPS spectra of PPor1-g-C_3_N_4_**





**Fig. S4.** XPS spectra of **PPor1-g-C_3_N_4_**.

**Section 3. The Tauc Plot of g-C_3_N_4_-N_3_**





**Fig. S5** The Tauc Plot of g-C_3_N_4_-N_3_.

**Section 4. The NLO performance of Por-g-C_3_N_4_**





**Fig. S6** The open aperture Z-scan tests of **Por-g-C_3_N_4_** in MMA (0.05 mg/mL).

**Section 4. References**

[1] F. D'Souza, G.R. Deviprasad, M.E. El-Khouly, M. Fujitsuka, and O. Ito (2001). Probing the donor-acceptor proximity on the physicochemical properties of porphyrin-fullerene dyad: "Tail-on" and "tail-off" binding approach. *J. Am. Chem. Soc.* 123, 5277-5284. doi: 10.1021/ja010356q

[2] J. Gao, Y. Zhou, Z. Li, S. Yan, N. Wang, and Z. Zou (2012). High-yield synthesis of millimetre-long, semiconducting carbon nitride nanotubes with intense photoluminescence emission and reproducible photoconductivity. *Nanoscale* 4, 3687-92. doi: 10.1039/c2nr30777d

[3] M. Jahan, Q. Bao, J.-X. Yang, and K.P. Loh (2010). Structure-Directing Role of Graphene in the Synthesis of Metal−Organic Framework Nanowire. *J. Am. Chem. Soc.* 132, 14487-14495. doi: 10.1021/ja105089w

[4] X. Liao, F. Wang, Y. Wang, W. Wei, Z. Xiao, H. Liu, Q. Hao, S. Lu, and Z. Li (2020). Functionalized g-C3N4 sheets assisted synthesis of growth-oriented MIL-88B-Fe with rod-like structure: Upgrading framework photo-catalytic performance and stability. *Appl. Surf. Sci.* 503, 144089. doi: [10.1016/j.apsusc.2019.144089](https://doi.org/10.1016/j.apsusc.2019.144089)
